# Study of Gravity Effects on Titanium Laser Welding in the Vertical Position

**DOI:** 10.3390/ma10091031

**Published:** 2017-09-08

**Authors:** Baohua Chang, Zhang Yuan, Haitao Pu, Haigang Li, Hao Cheng, Dong Du, Jiguo Shan

**Affiliations:** 1 State Key Laboratory of Tribology, Department of Mechanical Engineering, Tsinghua University, Beijing 100084, China; bhchang@tsinghua.edu.cn (B.C.); 15201518430@163.com (Z.Y.); puhaitaohehe@163.com (H.P.); 2 Aerospace Research Institute of Materials & Processing Technology, Beijing 100076, China; lhg703@sina.com (H.L.); chenghao611@126.com (H.C.)

**Keywords:** laser beam welding, positional welding, titanium alloy, porosity

## Abstract

To obtain satisfactory welds in positional laser beam welding, it is necessary to know how process parameters will influence the quality of welds in different welding positions. In this study, the titanium alloy Ti6Al4V sheets were laser welded in two vertical welding positions (vertical up and vertical down), and the appearance, porosity, strength, and ductility of the laser joints were evaluated. Results show that undercuts of the vertical up welds were greater than that of vertical down welds, while the porosity contents were much higher in vertical down welds than that in vertical up welds. When welding with a higher heat input, the vertical up welding position resulted in poor weld profiles (undercuts and burn-through holes), whereas the vertical down welding position led to excessive porosity contents in welds. Both severe undercut and excessive porosity were detrimental to the tensile properties of the welds. Weld appearance was improved and porosity contents were reduced by using a lower heat input, achieving better weld quality. Therefore, it is suggested that process parameter settings with relatively high laser powers and welding speeds, which can result in lower heat inputs, are used when laser welding the Ti6Al4V titanium alloys vertically.

## 1. Introduction

Nonferrous alloys, such as titanium alloys and aluminium alloys have been increasingly used in the aerospace and automobile industries because of their high specific strengths and, therefore, high fuel efficiency [1]. Meanwhile, the laser beam welding process has been increasingly adopted in manufacturing various engineering structures because of low distortion, high precision, and high productivity [2]. The structures to be welded may have complex designs and consist of welds of complex shapes. For such welds, different welding positions are required when the space orientations of the welds vary during laser welding, and the laser welding parameters need be adjusted accordingly for varying welding positions to achieve optimal welding quality. Hence, it is crucial to learn about how the welding process and welding quality can be affected by the different welding positions in laser welding.

Studies on the laser welding of high-strength steel sheets showed that using a horizontal welding position was helpful in mitigating the defects of undercut and sagging, which are commonly encountered when welding in a flat welding position [3]; moreover, higher laser power was required for a horizontal welding position than for a flat welding position [4]. Numerical analyses using computational fluid dynamics (CFD) codes showed that gravity had no noticeable influence on the dimensions and shapes of the keyholes and weld pools when laser welding mild steel sheets in eight typical welding positions; different porosity amounts, however, were revealed for welds of different welding positions [5]. In both mild steel and titanium alloy laser welds, different distributions and porosity amounts were found for different welding positions: the pores were distributed above the centreline in horizontal welds, but were along the centreline in flat welds. Moreover, the porosity amount in horizontal welds was greater than that in flat welds, and the excessive porosity in welds could have deteriorative effects on the weld strengths [4,6]. 

Laser welding of titanium alloys has been studied ever since the birth of the laser in 1960s. The laser beams used included both the early stage CO_2_ laser, the Nd:YAG laser [7,8,9,10,11,12], and the more recently developed fibre laser and disc laser [13,14,15]. Experimental development and numerical modelling were carried out to study how the laser welding parameters could influence bead morphology, joint microstructure, defects formation, and mechanical behaviour. In recent years, laser beams have even been employed to join dissimilar materials, such as titanium alloy to aluminium alloy [16,17]. Significant progress has already been made, based on the work already performed. However, existing research on the positional laser welding of titanium alloys is still very limited. Although the flat and horizontal laser welding of a titanium alloy has already been studied [6], vertical welding positions haven’t been addressed to date. 

Considering that the laser welding of titanium alloys in vertical positions is sometimes inevitable, this paper aims to investigate the laser welding of a titanium alloy (Ti6Al4V) in two vertical welding positions (vertical up and vertical down), and to reveal the influences of welding positions and laser welding parameters on the laser weld quality. 

## 2. Experimental Procedures

This study used titanium alloy sheets of Ti6Al4V, which had a thickness of 3 mm. Table 1 lists the chemical compositions of the alloy in addition to its basic mechanical properties. The sheets were cut into workpieces with dimensions of 250 mm × 150 mm × 3 mm. Laser welding was implemented using a 6 kW IPG Yb-fibre laser. The core diameter of the delivery optic fibre was 0.2 mm; the collimating and focusing lenses had focal lengths of 100 mm and 300 mm respectively. The laser beam width that resulted was 0.6 mm. 

**Figure 1:**
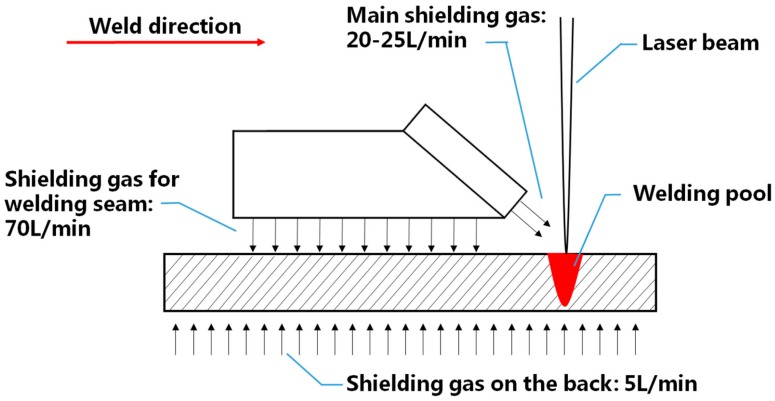
The shielding scheme adopted in this study.

Because titanium alloys are very sensitive to oxidation at high temperature, special measures were taken to protect the workpieces. The shielding scheme adopted in this study is shown in Figure 1. It can be seen that three shielding gas flows were adopted to protect the weld pool, the high-temperature metals that had just solidified, and the underside of the workpieces. High purity (99.998%) argon was used for all laser welding experiments, and the corresponding rates for the three flows were 20 L/min, 70 L/min, and 5 L/min, respectively.

**Figure 2:**
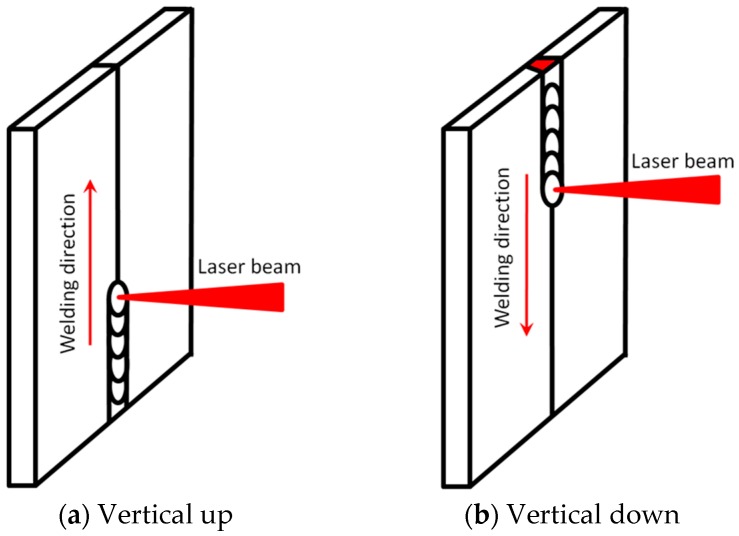
Schematic diagrams of the vertical welding positions investigated.

Figure 2 shows the two vertical welding positions, i.e., vertical up and vertical down, employed in the present study, and Table 2 presents the laser welding process parameters used to produce butt laser welds. The focal positions of the laser beam were the same for all welding experiments, with a defocusing distance of zero, i.e., the focal point was at the top surface of the workpieces. 

After welding, the bead appearance, inner porosity, and mechanical properties of welds were examined to evaluate the quality of the laser joints obtained. X-ray radiography was firstly used to detect the pores in the welds. Then, metallographic samples were prepared by grinding, polishing, and etching in a solution (2 mL HF + 10 mL HNO_3_ + 88 mL water), based on which the weld bead appearance, undercuts, and microstructures were examined under an optical microscope. Afterwards, three mechanical testing specimens were prepared for each welding parameter setting, which were then tested under static tensile conditions. Fracture surfaces after tests were analyzed using a scanning electron microscope. With all the results obtained, the correlations between the bead appearance, inner porosity, and tensile behaviour were analysed comprehensively.

## 3. Results

### 3.1. Appearance of Laser Weld Beads

**Figure 3:**
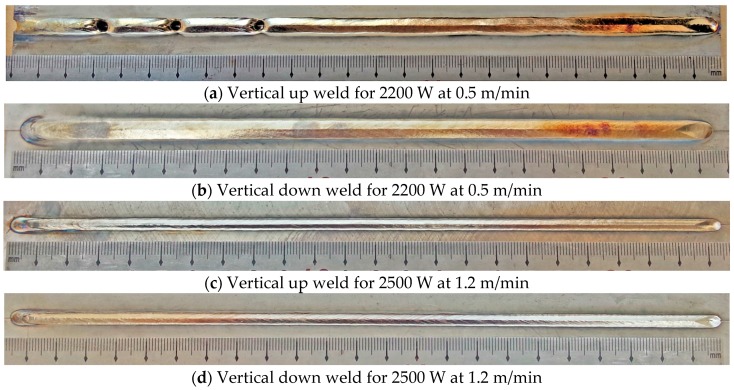
Appearances of butt weld beads made using different welding positions and process parameters.

Full penetrations were achieved in both vertical up and vertical down welding positions for all of the welding conditions listed in Table 2. Weld beads that appeared good and had consistent widths were obtained, except for the vertical up weld produced using lower laser power at slower welding speed (2200 W, 0.5 m/min), in which burn-through holes and inconsistent widths could be seen along the weld bead, as shown in Figure 3. 

**Figure 4:**
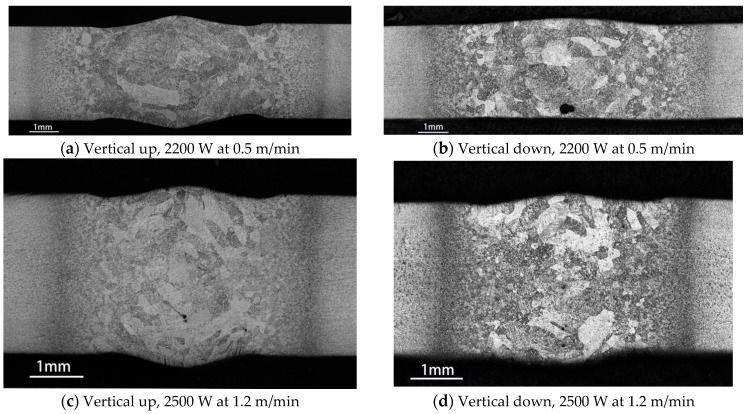
Laser bead shapes for different welding positions and process parameters.

The laser weld bead profiles obtained using four welding parameter settings are given in Figure 4, based on which the weld undercuts were measured, as listed in Table 3. It can be found that for the same welding parameters the vertical up welding will lead to larger undercuts than the vertical down welding. The undercuts decrease when increasing heat input from 125 kJ/m (2500 W at 1.2 m/min) to 275 kJ/m (2200 W at 0.5 m/min). An exception is the root undercut of vertical up welds, which increases when increasing the heat input as a result of the inconsistent root weld bead surface along the weld length. 

### 3.2. Porosity in Welds

**Figure 5:**
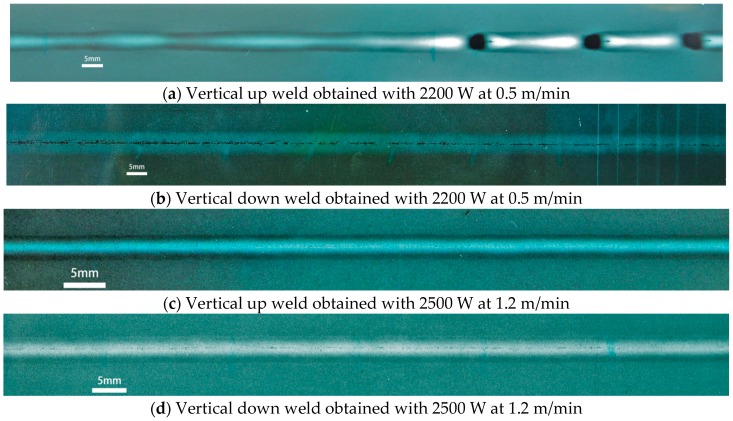
X-ray scanning results for the four laser butt welds.

Porosity in the welds was examined with X-ray radiography and the results are shown in Figure 5. These found that when laser welding used the higher heat input of 275 kJ/m (2200 W at 0.5 m/min), no pores were detected in the vertical up weld (Figure 5a). In contrast, severe chain porosity (lots of pores distributed in a line and very close to each other) was detected along the centerline of the vertical down weld (Figure 5b). 

When welding used the lower heat input of 125 kJ/m (2500 W at 1.2 m/min), a few small pores existed in the vertical up position weld (Figure 5c), indicating an increase in porosity due to the decrease in heat input. In the vertical down position weld, however, although chain porosity still existed (Figure 5d), it was much finer than that in the weld made using the higher energy input (275 kJ/m). 

**Figure 6:**
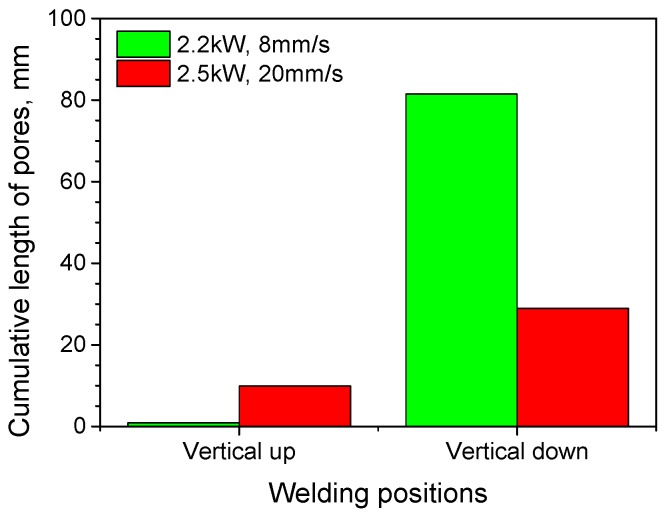
Cumulative lengths of pores under different welding conditions.

The total lengths of pores in the welds were measured for the four welding cases, and are shown in Figure 6. For each weld, the diameters of pores were measured over a weld length of 100 mm. It can be seen that the total lengths of pores in vertical up welds are much shorter than those in vertical down welds. For the vertical up position, the total length of pores was greater when welding used the lower heat input (2500 W, 1.2 m/min) than that using the higher heat input (2200 W, 0.5 m/min); while for the vertical down position, the total length of pores was notably decreased for the decreased energy input (corresponding to the higher laser power and traveling speed). 

### 3.3. Tensile Properties of Laser Weld Joints

For both vertical up and vertical down positions, the butt weld joints of the titanium alloy fractured through the base metal when the laser heat input was lower (2500 W and 1.2 m/min) in tensile testing, while the fracture occurred in the joint zone, which consisted of weld metal and the heat affected zone (HAZ), for those joints produced with higher energy input (2200 W and 0.5 m/min). 

**Figure 7:**
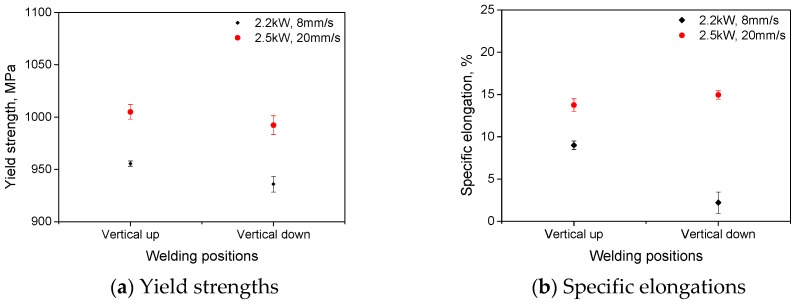
Tensile mechanical properties of laser joints made using different welding conditions.

The yield strengths and ductility (in terms of specific elongation) of the laser welds are shown in Figure 7. When welding used laser power of 2500 W and traveling speed of 1.2 m/min, comparable yield strengths (about 1000 MPa) and specific elongations (about 13.5%) resulted for vertical up welds and vertical down welds. For these welds, the cracks initiated and propagated within the base metal during tensile tests, and should therefore have led to basically the same yield strengths. When welding used lower laser power of 2200 W at lower traveling speed 0.5 m/min (resulting in a higher input), the yield strengths for both vertical up and vertical down welds decreased by about 50 MPa. For the vertical up position welds, the specific elongations decreased slightly with increasing heat input. By contrast, the specific elongations for the vertical down position welds decreased dramatically to only about 2.5% for the increased heat input. 

## 4. Discussion

### 4.1. Influence of Gravity on Formation of Defects

#### 4.1.1. Influence of Gravity on Bead Appearance

Examination over the bead appearances showed that burn-through holes existed in the vertical up position welds if the higher heat input of 275 kJ/m (2200 W laser beam power at 0.5 m/min welding speed) was used, which was absent when welding in the vertical down welding position. Furthermore, laser welding with a lower heat input of 125J/s (2500 W laser beam power at 1.2 m/min) could avoid the formation of burn-through holes.

**Figure 8:**
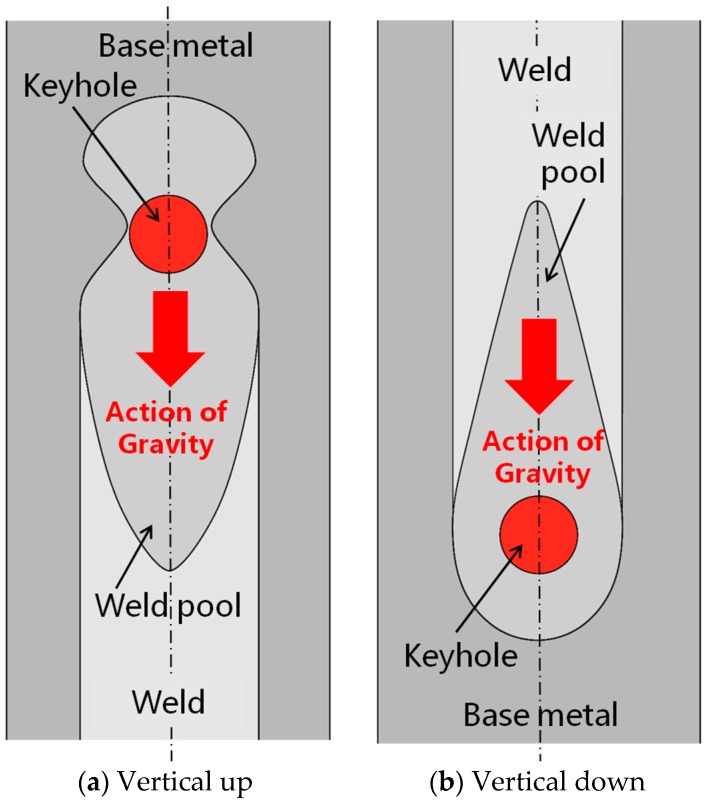
Schematic representations of the actions of gravity on molten metal in different welding positions.

For vertical up welding position (shown in Figure 8a), the downward fluid flow of molten metal behind the keyhole was enhanced under the action of gravity. This part of the molten metal tends to flow away from the keyhole and towards the rear part of the weld pool. Such downward flow, when becoming excessively severe due to too much heat input, will enlarge the keyhole and cause separation of the molten metal behind the keyhole from that ahead of the keyhole, and then result in a burn-through hole. For vertical down welding position, the molten metal behind the keyhole is also driven by gravity to flow downwards. However, in this case, the action of gravity was towards the keyhole (the front part of weld pool). Therefore, no separation of molten metal will occur, and no burn-through hole is formed, as shown in Figure 8b. 

The intermittent formation of burn-through holes during vertical up laser welding process may render the weld pool very dynamic and unstable. This accounts for the inconsistent bead profiles (undercuts and widths) along the length of vertical up welds, which were absent in vertical down welds. 

#### 4.1.2. Influence of Gravity on Formation of Pores

**Figure 9:**
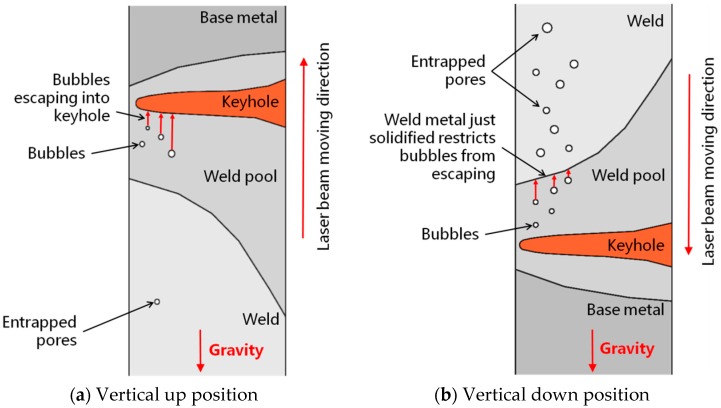
Movement of gas bubbles and voids in two welding positions.

X-ray examination results of porosity showed that the vertical up welding position led to less pores in laser welds than the vertical down welding position. During laser welding, gas bubbles or voids may form as a result of collapsing keyhole or turbulent fluid flow of molten metal in the weld pool. If there are routes available for theses bubbles and voids to escape, and the molten metal remains there long enough, the bubbles and voids will be able to leave the weld pool and won’t form pores in welds. Otherwise, if the escape routes are not available and/or the solidifying time is too short, the bubbles and voids will be retained in welds and form pores. For vertical up and vertical down welding positions, although the dimensions and solidifying time of weld pools can be similar when laser welding with the same welding parameters, there exist totally different escape routes. For vertical up welding position (Figure 9a), the voids or gas bubbles formed will move upwards in the weld pool in response to the buoyancy, similarly to that revealed using a transmission X-ray imaging system in the laser welding of other materials, such as aluminum alloys and steels [18,19]. Some of these voids and bubbles will move out of the weld pool through the interface between the weld pool and keyhole before the metal solidifies, while others cannot move out of the weld pool and form pores in the solidified welds. For vertical down welding position (Figure 9b), the voids or gas bubbles formed also buoy upwards. However, in this welding position the direction of movement of these bubbles/voids is opposite to the keyhole, and the upper edge of the molten metal is constrained by the recently solidified weld metal rather than the free keyhole space in vertical up welding. Under such conditions, there are no routes for bubbles/voids to escape and almost all of them are entrapped in the solidified weld metal. Consequently, a large number of pores exist within the vertical down welds, as shown in Figure 6.

Besides the welding positions, the final amount of porosity in laser welds can also be influenced by laser welding parameters in two basic ways. First, the laser parameters used will affect the stability of the fluid flow and keyhole, and then the amount of voids and bubbles formed in laser welding. As indicated by CFD modeling, the molten metal in weld pool is more unstable when laser welding with lower power and corresponding lower welding speed, which may then cause a larger number of pores [20]. By contrast, the fluid flow in the weld pool is less unstable for relatively higher laser beam powers in combination with faster traveling speeds, resulting in fewer pores. This explains why the porosity was much lower when higher power and welding speed were used in vertical down welding (as shown in Figure 6), in which all the bubbles formed in weld pools were retained in the welds. Second, the laser parameters may affect the size of the weld pools, and then the time allowed for bubbles to buoy and escape. Higher heat input will result in a larger weld pool, so the bubbles have a longer time to move upwards and more bubbles can move out of the molten metal before it solidifies. By contrast, lower heat input will produce a smaller weld pool, shorter solidification time, and more pores entrapped in the weld pool. This explains why for vertical up welds, which with an escape route available for bubbles as shown in Figure 9a, the total length of pores was increased by using a lower laser heat input, i.e., a slightly higher laser power but much faster traveling speed, as indicated by Figure 6. 

### 4.2. Influence of Defects on Fracture Behavior

**Figure 10:**
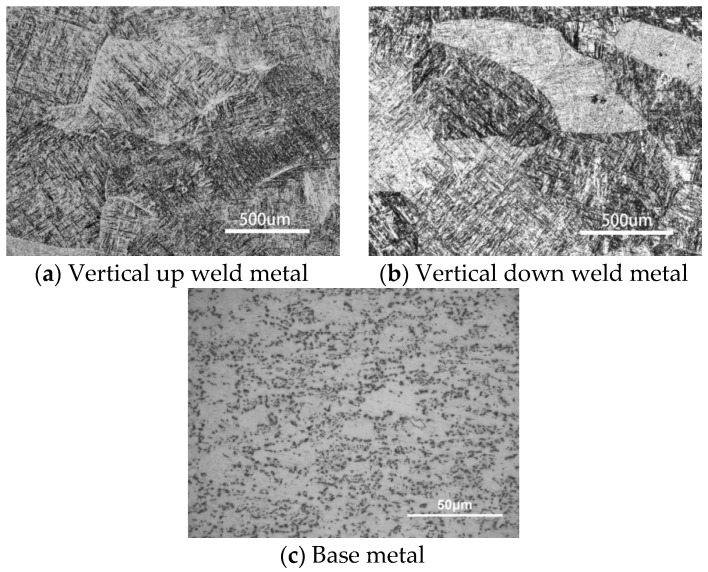
Microstructures of base metal and weld metals.

Experiment results show that, for both vertical up and vertical down positions, all welds produced with lower heat input (2500 W at 1.2 m/min) fractured through base metal, and had higher yield strengths and better ductility than those produced with higher heat input (2200 W at 0.5 m/min), for which the fracture occurred not through the base metal but through the joint zone consisting of the weld metal and heat affected zone (HAZ). Such a deterioration of mechanical properties can partially be attributed to the coarse martensite microstructure in the joint zone, as shown in Figure 10a,b for vertical up and vertical down welds respectively. The coarse martensites were formed by heat input that was too high and had lower fracture toughness than the original (α+β) phases in the Ti6Al4V parent material, as shown in Figure 10c, in which the white region is α phase and the black region is intergranular β phase. 

**Figure 11:**
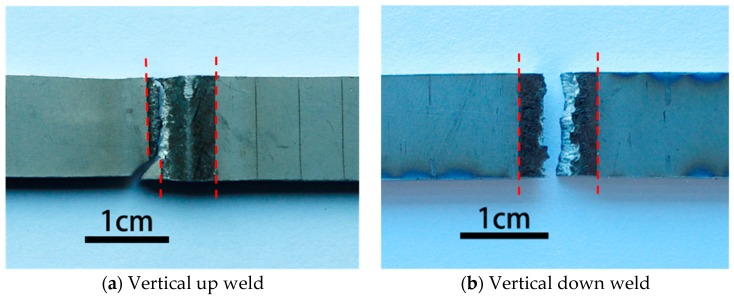
Fractured specimens of (**a**) vertical up weld and (**b**) vertical down weld with higher heat input (2200 W, 0.5 m/min).

**Figure 12:**
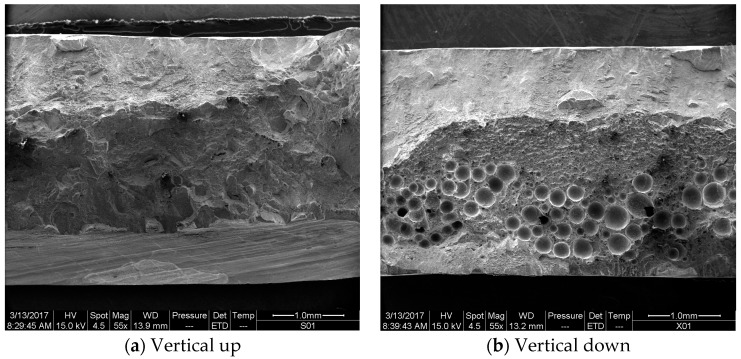
Fractographs of (**a**) vertical up weld and (**b**) vertical down weld with the higher heat input (2200 W, 0.5 m/min).

For vertical up position welds fabricated with 2200 W laser power and 0.5 m/min welding speed (the higher heat input condition), the crack initiated from and propagated through the boundary between the base metal and weld metal during the static tensile tests, as shown in Figure 11a. This should result from both the weakened weld metal and the severe weld undercut (listed in Table 3). The undercut can cause high stress concentration and then initiation and propagation of the crack in a tensile test. By contrast, for the corresponding vertical down weld, the crack initiated from and propagated through the centerline of the welds, as shown in Figure 11b. The undercut for this weld was the minimum among the four welding conditions. For this reason, the stress concentration was not severe, and the crack no longer initiated and grew through the edges of the weld bead. Instead, the crack initiated from the weld center, where a great number of pores were entrapped and chain porosity formed (as shown in Figure 5). The existence of such chain porosity made the initiation and propagation of cracks easier, which therefore deteriorated the strength and toughness of the welds notably (as demonstrated in Figure 7). Figure 12 presents the fracture surfaces of a vertical up weld and a vertical down weld, both of which were laser welded using the higher heat input of 275 kJ/m (laser power: 2200 W; traveling speed 0.5 m/min). No porosity is present in the vertical up weld (Figure 12a), while lots of pores can be seen in the vertical down weld (Figure 12b). 

## 5. Conclusions

In this paper, titanium alloy Ti6Al4V was laser welded in vertical positions, and the effects of gravity were studied on the weld bead profile and inner porosity, in addition to their correlations with the mechanical properties of the laser welds that resulted. The conclusions of the work can be summarized as follows:

(1) The undercuts of vertical up welds were greater than those of vertical down welds for the same welding parameters in this study. Burn-through holes and inconsistent weld bead appearance formed when vertical up welding using the higher laser energy input.
(2) The vertical down position could lead to more pores in laser welds than the vertical up position. Welding with the higher laser power at higher speed could reduce the porosity in vertical down welds.
(3) Both undercut and porosity were detrimental to the tensile mechanical properties of welds when the higher heat input was used. The vertical up weld fractured through the greatest undercut, while the vertical down weld fractured through the most severe porosity.
(4) Irrespective of welding positions studied, all laser joints welded using the lower energy input failed from the base metal, and had higher yield strengths and better ductility than those welded using the higher energy input, which failed from the weld metal.
(5) For both vertical up and vertical down welding, it is suggested that relatively high laser powers in combination with high travelling speeds are employed for better welds.

## Figures and Tables

**Table 1:** Chemical composition and mechanical properties of Ti6Al4V titanium alloy.

Chemical Composition									Mechanical Properties		
Elements	Al	V	Fe	C	N	H	O	Ti	σ_y_/MPa	σ_b_/MPa	δ_5_/%
Content, wt %	5.8	4.0	0.2	0.05	0.03	0.011	0.19	Bal.	955.2	1018.3	15.1

**Table 2:** Process parameters settings used in laser welding experiments.

Welding Positions	No. of Butt Welds	Laser Beam Power, W	Traveling Speed, m/min	Heat Input, kJ/m
Vertical up	U01	2200	0.5	275
	U02	2500	1.2	125
Vertical down	D01	2200	0.5	275
	D02	2500	1.2	125

**Table 3:** Undercuts of welds produced with different welding conditions (unit: µm).

Welding Position	No. of Butt Welds	Face Undercut	Root Undercut
Vertical up	U01	77.2	119.9
	U02	116.2	47.0
Vertical down	D01	0.0	0.0
	D02	38.5	13.7
